# Using Play to Improve Infant Sleep: A Mixed Methods Protocol to Evaluate the Effectiveness of the Play2Sleep Intervention

**DOI:** 10.3389/fpsyt.2018.00109

**Published:** 2018-04-17

**Authors:** Elizabeth Keys, Karen M. Benzies, Valerie Kirk, Linda Duffett-Leger

**Affiliations:** ^1^Faculty of Nursing, University of Calgary, Calgary, AB, Canada; ^2^Alberta Children’s Hospital/University of Calgary, Calgary, AB, Canada

**Keywords:** infant, sleep, night wakings, mothers, fathers, parent–child interactions

## Abstract

**Background:**

One in four Canadian families struggle with infant sleep disturbances. The aim of this study is to evaluate Play2Sleep in families of infants with sleep disturbances. In addition to parental education on infant sleep, Play2Sleep uses examples from a video-recorded, structured play session with mothers and fathers separately to provide feedback on parent–infant interactions and their infant’s sleep-related social cues. The quantitative phase will answer the research question: Does one dose of Play2Sleep delivered during a home visit with mothers and fathers of infants aged 5 months reduce night wakings at age 7 months? The qualitative phase will answer the research question: What are parental perceptions of family experiences, processes, and contexts related to Play2Sleep and infant sleep? The overarching mixed methods research question is as follows: How do parental perceptions of family experiences, processes, and contexts related to infant sleep explain the effectiveness of Play2Sleep?

**Method and analysis:**

An explanatory sequential mixed methods design will be used. In the quantitative phase, a randomized controlled trial and RM-ANOVA will compare night wakings in infants whose parents receive Play2Sleep versus standard public health nursing information. Sixty English-speaking families (mothers and fathers) of full-term, healthy, singleton, 5-month-old infants who perceive that their infant has sleep disturbances will be recruited. The primary outcome measure will be change in the number of night wakings reported by parents. The qualitative component will use thematic analysis of family interviews to describe parental perceptions and experiences of infant sleep. Mixed methods integration will use qualitative findings to explain quantitative results.

**Discussion:**

Play2Sleep is a novel approach that combines information about infant sleep with personalized feedback on parent–infant interactions and infant cues. Including fathers and mixed methods should capture complex family experiences of infant sleep disturbances and Play2Sleep. If effective, Play2Sleep has possible application for preventing infant sleep disturbance and tailoring for other populations.

**Clinical Trial Registration:**

www.ClinicalTrials.gov, identifier: NCT02742155. Registered on 2016 April 23.

## Introduction

Approximately one in four Canadian families experience infant sleep disturbances (1). Infant sleep disturbances are associated with parental feelings of guilt and helplessness, as well as maternal depression and anxiety (2–5). Parents may bed-share with their infants in response to infant sleep disturbances despite awareness of safe sleep recommendations that discourage this practice (6, 7). Furthermore, infant sleep disturbances are associated with increased infant stress hormones (8) and may predict socio-emotional difficulties (9). Assisting families in managing infant sleep disturbances may improve family health, as well as support child development. Time constraints and lack of provider confidence in being able to address sleep concerns may hinder primary health care providers’ ability to address sleep concerns during primary care visits (10). The proliferation of privatized and unregulated sleep consultants (11) highlights a noteworthy gap in Canada’s public health care system.

In a Cochrane review of postnatal parental education, parental education was shown to be effective in improving the amount of nighttime infant sleep duration by 29 min; however, similar improvements in infant crying were not observed (12). Other individual trials provide further evidence that parental education on infant sleep can be an effective means for changing sleep-related parenting practices (13) and improving infant nighttime sleep consolidation while decreasing parental perception of night waking severity (14). Behavioral-based sleep interventions improve sleep in over 80% of children under age 5 years (15). Yet, in a Canadian community-based survey, less than half of parents reported they were able to successfully manage their infant’s sleep disturbances using behavioral-based sleep strategies (16). Furthermore, parents may experience reservations and practical challenges when applying behavioral-based approaches (17, 18). Moreover, despite evidence that higher father engagement may mitigate maternal stress associated with infant sleep disturbances (19) and reduce infant night wakings (20), including fathers in infant sleep intervention research is limited.

Addressing only sleep-related parenting behaviors and interactions may be ineffective in improving broader parenting difficulties that may underlie infant sleep disturbances (21, 22). When examining links between maternal sensitivity and infant sleep, researchers have found that maternal sensitivity was positively linked to subsequent, but not concurrent (23), sleep consolidation (24, 25). Mothers observed to be more sensitive during an unstructured play session, according to the adult sensitivity scale of the Child–Adult Relationship Experimental Index, reported better perceived sleep quality in their 7- to 18-month-old infants (26). However, parent–child interactions are influenced by more than only maternal sensitivity and it may be important to recognize the dyadic nature of parent–child interactions. For instance, Scher (23) found that infants who were highly responsive in play interactions had more frequent night wakings than infants who were not as responsive. Some researchers have instead examined how infant sleep may be related to emotional availability, a dyadic concept that considers the emotional quality of parent–child interactions and includes sensitivity. Findings from these researchers demonstrate a positive association between greater emotional availability at bedtime and fewer night wakings (27) and more rapid increases in the amount of nighttime sleep during the first 6 months of life (28).

Treyvaud and colleagues (29) found mothers who reported poorer sleep quality themselves were observed to have greater difficulty recognizing and responding to their infant’s subtle and potent social cues. Over the duration of a 5-day residential parenting program, this sample of mothers was observed to have improvements in overall parent–child interactions, including contingent responses, and perceived that their child’s behavior difficulties, 80% of which were related to sleep issues, had decreased in frequency and severity (29). Another intervention with parents of maltreated toddlers aimed at improving the parent–child relationship by promoting responsive care using a series of video feedback sessions resulted in a reduction in sleep disturbances (30). However, researchers have not examined this relational approach in an otherwise healthy community-based population experiencing sleep disturbances. Thus, promoting positive parent–child interactions in a manner that enhances parental ability to identify infant sleep-related social cues represents a novel strategy that could be used to complement educational approaches to more effectively support families challenged with infant sleep disturbances.

Parent–child interactions influence the architecture of children’s growing brains, as well as developmental outcomes (31, 32). High quality parent–child interactions occur with sensitive and effective parental behaviors that are contingent upon the child’s clear and responsive verbal and non-verbal communication cues (33, 34). Parent coaching can improve the quality of parent–child interactions. This type of coaching is closely aligned with the skill set of registered nurses, such as expertise in relational communication, knowledge of healthy early childhood development, and proficiency in strengths-based approaches (35, 36). Providing personalized feedback on self-modeled, video-recorded, structured parent–infant interactions is a feasible and effective strategy for improving parental interactions with preterm and term infants (37–40).

The proposed nursing theory described by Keys and Benzies (41) forms the theoretical underpinnings of this study. Informed by Bronfenbrenner’s ecological theory (42, 43) and Barnard’s Model of parent–child interactions (44), Keys and Benzies guide nurses to consider parental knowledge and beliefs about infant sleep with the family’s synchrony, not only relating to sleep but also the family’s ability to engage in effective parent–infant interactions. As such, nurses are encouraged to enhance parental knowledge related to infant sleep, as well as the underlying foundational familial interactions that support parental ability to apply knowledge relating to infant sleep during daily and foundational parent–infant interactions. Play2Sleep is an intervention that uses personalized feedback of structured parent–infant play sessions. This approach could help address broader parenting difficulties that may underlie infant sleep disturbances by enhancing parental ability to identify and respond appropriately to their infant’s specific sleep-related and social cues. This study extends previous self-modeled, video-recorded interaction guidance interventions and aims to evaluate Play2Sleep to improve infant sleep. This protocol was prepared in accordance with the SPIRIT guidelines (45), as well as the TIDieR checklist and guide (46) for reporting of interventions.

The overarching mixed methods research aim is to understand how parental perceptions of family experiences, processes, and contexts related to infant sleep explain the effectiveness of Play2Sleep. The objective of the quantitative phase is to compare the effect on infant sleep of one dose of Play2Sleep delivered during home visits with mothers and fathers of 5-month-old infants with sleep disturbances against standard infant sleep education only. The primary hypothesis is that Play2Sleep will decrease the number of night wakings at the age of 7 months. The objective of the qualitative phase is to describe parental perceptions of family experiences, processes, and contexts related to Play2Sleep and infant sleep.

## Materials and Equipment

### Study Design

**Figure 1:**
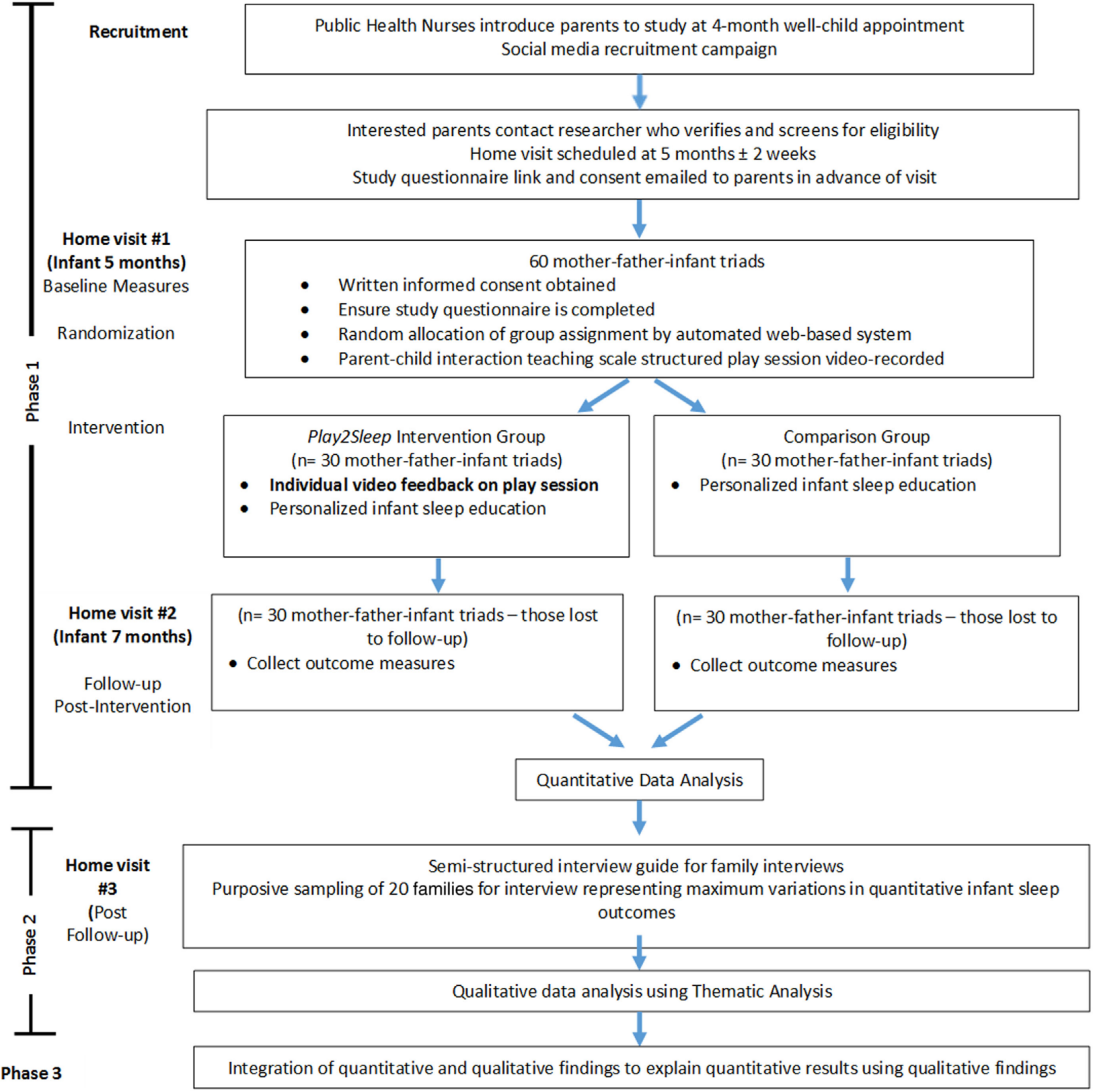
Flow chart of the study.

This study will employ a mixed methods, explanatory sequential design to evaluate the Play2Sleep intervention, with priority given to the quantitative phase (Figure 1). Phase 1 is a single center, prospective, randomized, controlled, parallel group, two arm, superiority trial with a 1:1 allocation ratio (ClinicalTrials.gov, identifier NCT02742155). Baseline data collection and intervention will occur when the infant is 5 months of age (±2 weeks), with outcomes measured when the infant is 7 months of age (±2 weeks). During Phase 2, interviews will be conducted with a subset of participating families (mothers and fathers together) about their perceptions and experiences related to their infant’s sleep. The interviews will occur after the outcome home visit takes place, and analyzed using thematic analysis. Phase 3, the integration phase, will focus on explaining the quantitative results from Phase 1 using the qualitative findings from Phase 2.

#### Rationale for Study Design

Researchers are increasingly using qualitative research to facilitate the interpretation of randomized controlled trial findings (47). The quantitative phase will provide evidence relating to the measurable impact of Play2Sleep, while the qualitative phase will provide crucial information that will contextualize the outcomes of Play2Sleep and increase understanding of the processes that contribute to its effectiveness. The integration of qualitative and quantitative findings will be used to explain the measureable impact of Play2Sleep using perceptions and experiences described by parents.

### Participants

The study will take place in a large urban center in Western Canada (Calgary, AB, Canada). Public health nurses will introduce potential participants to the study during 4-month well-baby clinics at eight urban-zone local community health centers. In addition to recruitment posters and postcards placed at each of the community health centers, researchers will use a social media campaign to promote the study on Facebook and Kijiji. At the community health centers, interested families will receive a card with a brief description of the study and public health nurses will request written permission from the parents to be contacted by the researcher. Upon contact with interested families, the researcher will follow a telephone script to (1) screen parents for eligibility, (2) obtain verbal consent from both parents to participate, (3) collect contact information, and (4) schedule the first home visit. Parents who are ineligible for the study will be offered standard public health information on infant sleep and provided with a list of community resources to which they can self-refer.

Participants (mothers and fathers) will be eligible if they meet the following inclusion criteria: (1) are cohabiting mothers and fathers of full-term, healthy, singleton, 4-month-old infants; (2) are first-time parents; (3) are able to read, write, and speak English; (4) perceive that their infant has sleep disturbances; (5) have an infant who has greater than 3 night wakings per night and/or is awake greater than 60 min during the night, and/or has less than 9 h total day and nighttime sleep. Infants meeting one or more of these three objective sleep criteria were considered to be poor sleepers by Sadeh (48) in the development of the original Brief Infant Sleep Questionnaire (BISQ). Participants will be excluded if there is a known or suspected medical or physiological cause of sleep disturbance in either parent or their infant.

### Sample Size

**Figure 2:**
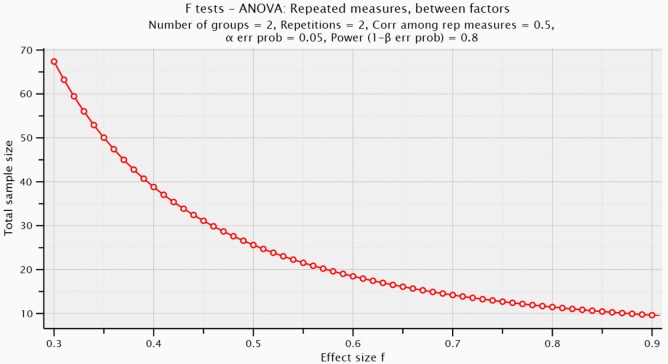
Effect size as a function of total sample size for power of 0.80.

**Figure 3:**
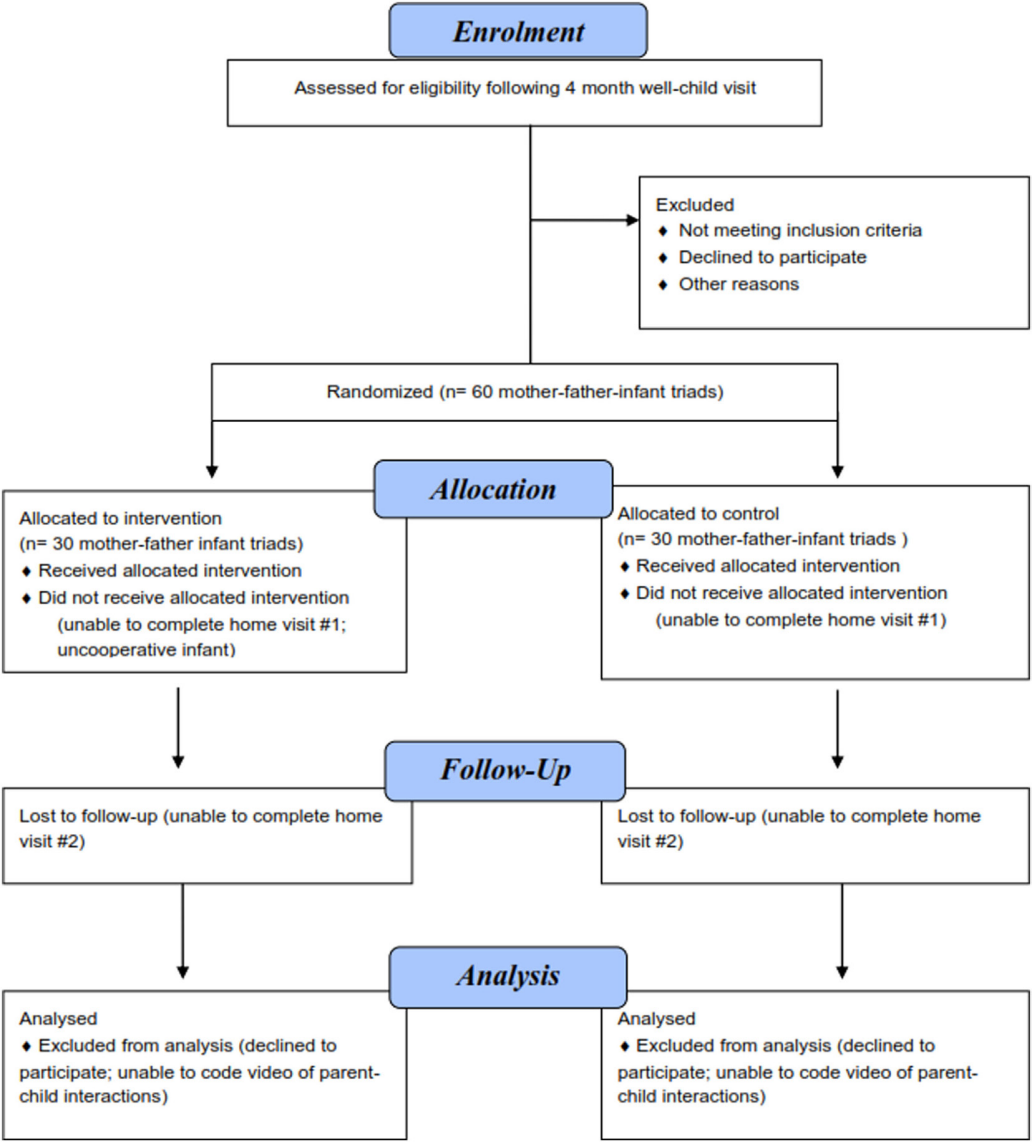
Proposed Consolidated Standards of Reporting Trials (CONSORT) flow diagram.

The effect size for the sample size calculation for the quantitative phase was estimated from research that examined brief sleep interventions with the number of night wakings as the primary outcome. A study of a brief internet intervention for families of young children up to age 3 years that provided a customized sleep program, with and without a standardized bedtime routine, found relatively large effect sizes (partial eta squared) of 0.442 and 0.441, respectively (49). In a narrower age group of infants 6–12 months, Hall and colleagues (50) found an effect size (Cohen’s *d*) of 0.64 for an intervention that included a 2-h teaching session and telephone support for parents. An *a priori* analysis was conducted in G*Power for repeated measures ANOVA, between factors (51). This calculation determined that a total sample size of 60 mother–father–infant triads (30 families in each group) would detect an effect size of 0.32 with a power of 0.80 at a 0.05 level of significance (Figure 2), which is below the effect size of the aforementioned studies (49, 50). Recruitment will be ongoing until complete data are collected from 60 families. Based on previous studies (37, 39), attrition is estimated to be very low (less than 5%) after the initial home visit. Figure 3 shows the proposed Consolidated Standards of Reporting Trials flow diagram.

For the qualitative component in Phase 2, Braun and Clarke (52) have recommended using sample sizes of 10–20 for a medium scope thematic analysis project. Because this thematic analysis is part of a larger mixed methods study and could, thus, be considered a project of medium scope, 20 families (10 from the experimental group and 10 from the comparison group) will be included in Phase 2.

### Trial Arms

#### Play2Sleep Experimental Group

The Play2Sleep intervention will be administered during a home visit. Mothers and fathers will be recorded separately while engaging in a structured play session with their infant that typically lasts 3–5 min, but no longer than 10 min, as per the Parent–Child Interaction Teaching Scale (PCITS) protocol (34). Immediately following the play session, a specially trained registered nurse (EK) will review the video recording with each parent separately, which will take about 15 min per parent. During this review, parents will receive 2–3 positive feedback comments on behaviors that promote overall interaction and child development, as well as 1–2 suggestions for areas of growth. To help parents address infant sleep concerns, this video feedback will also include the identification of real-life examples of the individual infant’s actual social and sleep-related cues, including both subtle and potent cues for engagement and disengagement, as described by NCAST Programs (34). According to the robust and well-defined NCAST classification system, infant cues can be broken down into engagement and disengagement cues (34). Engagement and disengagement cues are further broken down into subtle and potent cues. Both the subtle and potent disengagement cues are similar to cues that indicated drowsy and overtired infant states. The structured play session is used to elicit infant disengagement cues (both subtle and potent) to provide examples of the variety and range of cues that infants may use to indicate to their parents that they are needing a break soon, with sleep being an important and penultimate “break” from interaction. Particular attention will be focused on helping parents identify clusters of subtle and/or potent disengagement cues (Table 1) that signal the infant requires a break from the interaction, as well as additional indicators of the drowsy state (34). A handout distinguishing subtle and potent disengagement cues will be provided to parents to reinforce the information presented. Provided that the infant does not require care (feeding, diaper change, etc.) during the intervention, the entire play session and review will take approximately 20–30 min for each parent. Thus, the total play-related component of the Play2Sleep intervention, including the collection of the video-taped play interaction, takes approximately 50 min (5–10 min of play, plus 15 min of review, for each parent). During the video recording and review, the other parent will be asked to remain in another room out of visual and auditory contact with the interaction. At the end of the visit, the standard public health handouts on infant sleep will be reviewed with both parents together. These handouts include information on typical infant sleep patterns, including infant sleep cycles and sleep cues, as well as the importance of sleep associations and routines. Copies of these handouts are available online at: http://foothillsnetwork.ca/get-informed/helpful-links/. This discussion will take approximately 30 min, and it will be individualized to focus on the parents’ most pressing sleep-related concerns. The total home visit length for the Play2Sleep Experimental Group is approximately 1.5 h.

**Table 1:** Subtle disengagement, potent disengagement, and drowsy cues (34).

Subtle	Potent	Drowsy
Gaze aversion, looking away Hand-behind-head or ears Hand to mouth, eye, ear, or stomach Dull looking face/eye Head lowering Joined hands, self-clasp Lip compression, grimace, or pout Sobering, tongue show, ugh face Cling posture, diffuse body movements Facial grimace Frown or brow lowering Finger extension Yawn Rapid wrist rotation Wing palm	Back arching Halt hand Pushing or pulling away Crying, cry face Maximal lateral gaze aversion Overhand beating	Variable activity Irregular respirations Opens and closes eyes Eyes glazed, heavy-lidded look Delayed responsiveness

#### Comparison Group

The comparison group will also receive an intervention during a home visit. The comparison intervention consists only of the individualized review of the standard public health handouts on infant sleep with both parents, based on the parents’ most pressing sleep-related concerns. This discussion will take approximately 30 min. Although each parent in the comparison group will be video-recorded engaging in a play session to capture baseline and outcome parent–child interactions, they will not receive any feedback or review of the video recording. Instead of completing reviews of the video-taped play session, the home visitor proceeds directly to the discussion with both parents of the standard public health handouts on infant sleep. Thus, the comparison group home visit lasts approximately 1 h, which is about the same length of time for the intervention group.

#### Discontinuing or Modifying Allocated Interventions

Given the brevity of the intervention, situations that require discontinuing or modifying the allocated intervention are expected to be rare. In cases of family illness or acute parental mental or physical health concerns, the home visit will be either rescheduled or discontinued altogether, according to family preference. Families who discontinue will be offered a listing of local community health services to which they can self-refer.

#### Adherence

Strategies to increase adherence to study intervention protocols include the use of the same home visitor (Elizabeth Keys) for baseline home visits. As the intervention is concentrated in one home visit and relates to increasing parental ability to read and respond to their infant’s sleep and social cues, no additional adherence strategies will be used. Families will be allowed to contact the home visitor if they require clarification about the information provided during the home visit.

#### Concomitant Care

Families in both groups will not be restricted to using only the sleep strategies or suggestions received during the home visit. Rather, the baseline and outcome questionnaires will collect information about any additional strategies, resources, or services parents use.

#### Blinding

Given that the same home visitor will conduct both the experimental and comparison interventions, the home visitor cannot be blinded to group. Participants will be partially blinded by being informed that the research study is testing two different types of information about infant sleep during the home visit to see which one is superior. In addition, both groups will complete the PCITS protocol as an indicator of the quality of parent–child interactions, which requires parents to engage in a brief structured play interaction with their child. Blinding to group will occur during coding of parent–child interaction videos and data analysis. Given the brief and low-risk nature of the intervention, there are no anticipated circumstances under which unblinding will be required.

### Equipment

A PCITS kit,^1^ along with a digital video camera (Sony HDR-XR350 Handycam Camcorder) and adjustable tripod will be required for the home visits. In addition, a monitor (Sharp LC-13E1U television monitor) that connects to the video camera to play back the digitally recorded play sessions will be used. For the family interviews, a digital voice recorder (Sony IC recorder ICD-SX68) will be used to audio record the interviews.

### Measures

The primary infant sleep outcome will be the frequency of night wakings, as measured by the expanded version of the Brief Infant Sleep Questionnaire [BISQ (48, 53)]. The BISQ is not a psychometric scale, but a screening questionnaire that can be used in clinical practice and research. The expanded version of the BISQ is a multi-dimensional 32-item screening tool (53) developed from the well-established original 13-item tool (48, 54). The original and expanded BISQ both include items covering the domains of sleep ecology, infant sleep patterns, and caregiver perception of infant sleep. However, using the expanded version of the BISQ addresses limitations of previous studies where unspecific and incomplete information on daytime sleep, longest consolidated period of sleep, and sleep ecology, all of which may influence the families’ subjective perception of the presence of sleep problems (53). Given that the BISQ is a screening questionnaire and not a scale, Sadeh (48) did not provide a Cronbach’s α for the BISQ. Secondary outcomes for this study will include (1) nocturnal wakefulness, (2) sleep latency (the length of time it takes parents to put the infant to sleep in the evening, with responses captured as “less than 5 min,” “5–15 min,” “16–30 min,” “31–60 min,” and “more than 1”), (3) the longest consolidated sleep episode, and (4) parental perception of problematic infant sleep; these secondary outcomes will also be measured using the expanded BISQ. The expanded BISQ will be administered as part of a complete study online questionnaire package that includes questionnaires measuring covariates, in the 5–7 days before each of the baseline and outcome home visits. Outcome and covariate measures are presented in Tables 2 and 3. Demographic information, including (1) parental age, education, ethnicity, and income; and (2) infant age, birth order, and feeding type will be collected. At baseline and outcome, parents will be asked to describe the techniques, strategies, and resources that they use to promote infant sleep, which will be explored further during the qualitative interviews.

**Table 2:** Measure for primary (frequency of night wakings) and secondary (nocturnal wakefulness, sleep latency, longest consolidated sleep episode, and parental perception of problematic infant sleep) outcomes.

Measure	Description
Expanded Brief Infant Sleep Questionnaire [BISQ; (48, 53)]	32-item questionnaire of infant sleep patterns, sleep ecology, and caregiver perceptions; responses include numerical (time, frequency), categorical (sleep location, bedtime activities), and ordinal rankings (how much of a problem is your baby’s sleep). No composite score is calculated. High 3-week test–retest reliability for sleep duration and wakefulness, night wakings, sleep-onset time, and settling time (0.82–0.95; 48). Used to assess sleep interventions for infants 6–36 months with a parent-identified sleep problem and detect significant short- and long-term improvements in the number of infant night wakings and sleep latency (49, 55, 56). Used in an internet sample (*N* = 4,505) of Canadian and United States parents of infants aged 0–36 months (53) and an international sample (*N* = 29,287) of parent of infants aged 0–36 months (57). Night wakings correlated with actigraphy, *r* = 0.42, and daily logs, *r* = 0.83 (48). Frequency of night wakings accounted for 13.27% of variance in parental perceptions of severity of sleep problems (53). Differentiated between clinical and control infants with two BISQ items, frequency of night wakings and nocturnal sleep duration, with assignment of cases to the correct group 85% of the time (48). Average completion time is 6.21 min (53)

**Table 3:** Covariate measures.

Measure	Description
PCITS (34)	73-item binary measure of parent–child interactions on 4 parent (sensitivity to cues, response to distress, social-emotional, and cognitive growth fostering) and 2 child (clarity of cues and responsiveness to caregiver) subscales. Higher scores indicate higher quality interactions. Internal consistency for mothers, α = 0.53–0.87 (34) and fathers, α = 0.64–0.82 (58). Test–retest reliability (1 and 12 months) of 0.85 for caregiver and 0.55 for infant subscales (34). Concurrent validity with Home Observation Measure of the Environment scale (59). Predicts attachment, cognitive and language abilities (34, 60). Play session typically lasts 3–5 min
EPDS (61)	10-item measure of symptoms of depression, anxiety, and suicidal thoughts. Higher scores indicate more depressive symptoms. In mothers, correlated with Beck Depression Inventory second version, 0.82 (62). In fathers, EPDS was correlated with Center for Epidemiologic Studies Depression Scale, 0.62 (63). Internal reliability in mothers, α = 0.53–0.87 (62) and fathers, α = 0.87 (63); takes 3–5 min
IBQ-R very short form (64, 65)	37 statements of parental perception of infant temperament on 3 subscales: negative emotionality, positive affectivity, and regulatory capacity. Rated on a 7-point scale; higher scores indicate more difficult temperament. Internal reliability was 0.75–0.78 (65). Subscale test–retest reliabilities (2 and 12 months) from 0.64 to 0.88 (65). Mother–father inter-rater correlations from 0.28 to 0.61 for infants aged 4–6 months (65). Correlations with IBQ-R from 0.71 to 0.86; takes 12 min (65)
MCISQ (66)	20 items about parenting beliefs about infant sleep on 5 subscales: setting limits, anger, doubt, feeding, and safety; higher scores indicative of more negative concerns and doubts (66). Cronbach’s α = 0.82; 1-month test–retest = 0.81 (66). Mother–father correlations from 0.65 to 0.72 (67). Mothers’ scores (doubt) and fathers’ scores (anger) correlated with actigraphy (67). Overall score (mothers) and limit setting subscale (mothers and fathers) discriminated clinical and control groups (67); takes 5–10 min
DAS-4 (68, 69)	4 item measure of marital satisfaction; higher scores indicate stronger satisfaction. Internal consistency reliability (α = 0.84); temporal stability demonstrated using structural equation modeling over a 2-year period (69). Established discriminant validity for couple distress and predictive validity for couple dissolution (69). Used to assess relationship satisfaction in fathers (70), as well as in married and unmarried parents (71); takes 3–5 min
PSOC (72)	16-item measure with satisfaction, efficacy (72), and interest (73, 74) subscales; higher scores indicate higher parental competence. Internal reliabilities for satisfaction (0.75) and self-efficacy (0.76) subscales (72). Test–retest (6 weeks) for the total score and subscales ranged from 0.46 to 0.86 (72). Convergent with internalizing (−0.17 to −0.27) and externalizing (−0.10 to −0.31) behaviors on the Child Behavior Checklist (72) and marital satisfaction, 0.27 to 0.32 (75). Used to assess parental competence in mothers of children (0–4 years) with sleep problems (76); takes 3–5 min

*NOTE: Estimated time to complete questionnaire package is 36–57 min. PCITS, Parent–Child Interaction Teaching Scale; EPDS, Edinburgh Postnatal Depression Scale; IBQ-R, Infant Behavior Questionnaire—Revised; MCISQ, Maternal Cognitions about Infant Sleep Questionnaire; DAS-4, Brief version of the Dyadic Adjustment Scale; PSOC, Parental Sense of Competence*.

### Stepwise Procedure

#### Data Collection and Management

This community-based study will use both an online format and in-person home visits to collect baseline and outcome data (for a schedule of assessment, see Table 4) from both mothers and fathers separately. Families will be assigned a unique study identification number, which will be used to link mothers and fathers of the same family.

**Table 4:** Play2sleep study schedule of enrollment, interventions, and assessments.

Study period						
	Enrollment	Baseline	Randomization	Initial visit	Outcome visit	Family interview
Time point	*−t*2	*−t*1	0	*T*1	*T*2	*T*3
**Enrollment**						
Eligibility screen	X					
Informed consent	X (verbal)			X (written)		
Allocation			X			
**Intervention**						
*Play2Sleep Group*				X		
*Comparison Group*				X		
**Assessments**						
*Expanded BISQ*		X			X	
*PCITS*				X	X	
*EPDS*		X			X	
*IBQ-R very short form*		X			X	
*MCISQ*		X			X	
*DAS-4*		X			X	
*PSOC*		X			X	
*Interview*						X

*BISQ, Brief Infant Sleep Questionnaire; PCITS, Parent–Child Interaction Teaching Scale; EPDS, Edinburgh Postnatal Depression Scale; IBQ-R, Infant Behavior Questionnaire—Revised; MCISQ, Maternal Cognitions about Infant Sleep Questionnaire; DAS-4, Brief version of the Dyadic Adjustment Scale; PSOC, Parental Sense of Competence*.

Online study questionnaires, hosted on Qualtrics, will be used to collect the majority of data on outcomes and covariates at baseline, when the infant is aged 5 months, and at outcome, when the infant is aged 7 months. Qualtrics is a recognized and secure electronic survey system that houses data on secure Canadian servers. Parents (mothers and fathers) in both groups will each complete baseline questionnaires in the 7 days prior to the initial home visit. A link to the baseline questionnaires will be emailed to parents 5–7 days before the home visit, and an email reminder encouraging parents to complete the questionnaires prior to the home visit will be sent if necessary.

The first home visit will occur when the infant is 5 months old (±2 weeks), at a time that is convenient to the family. Benzies and colleagues (38) have demonstrated 5 months of age is a feasible time-point to begin a video self-modeled intervention. Scheduling the home visit when the infant is 5 months of age will allow sufficient time between referral to the study at the 4-month well-child clinic visit and scheduling of the first home visit. Scheduling the home visits within a 2-week window of 5 months provides a realistic timeframe to schedule visits based on availability of both parents, as well as being able to re-schedule visits if there are unanticipated events (i.e., infant illness). This narrow window minimizes maturation bias due to the rapid developmental changes that occur in infants at this age.

An automated randomization service (77) will generate the randomization sequence to allocate families to either the experimental or comparison group. Allocation concealment will be achieved because the randomization code will not be requested until the family enrolls in the study and parents complete the baseline questionnaire. After participants have completed the baseline questionnaire and immediately before the initial home visit, the home visitor will submit an online form requesting treatment allocation. A copy of the allocation will be emailed to the home visitor and stored in the study’s online Sealed Envelope Ltd. account.

At the start of the first home visit, participants will affirm their verbal consent (provided during the eligibility screening phone call) by providing written consent. After establishing initial rapport with the parents, the home visitor will ask each parent (mother and father) to teach their infant a unique age-appropriate play task, which is video-recorded by the home visitor to be coded later using the PCITS coding scheme (34). As per the PCITS manual, the video-taped structured teaching and play sequence lasts no longer than 10 min, typically spanning a 3- to 5-min timeframe (34). The order of the video-recordings will alternate between the mother and father to prevent ordering bias. Following the video-recorded structured play session, the home visitor will deliver either the Play2Sleep or comparison intervention. At the conclusion of the home visit, the home visitor will offer to schedule the outcome home visit.

The outcome home visit will occur when the infant is aged 7 months (±2 weeks). Seven months was selected because parents need time to practice and solidify new interaction patterns to translate into measurable effects on infant sleep patterns. Parents (mothers and fathers) in both groups will each complete outcome questionnaires in the 7 days prior to the second (outcome) home visit. During the outcome home visit, parents in both the experimental and comparison groups will again engage their infant in a different play task that is video-recorded for later coding. All families will be offered a listing of local infant sleep-related resources and services. Once data collection is completed, data will be downloaded, backed-up, and deleted from the Qualtrics account.

Following the home visits, a reliable coder who has achieved the required reliability of 90% with the PCITS training videos and who is blind to group allocation will code the videos. Coding of video-recorded interactions occurs at a later time and typically takes 30 min. A random selection of 10% of the videos will be re-scored to assess intra-rater reliability. Another reliable PCITS coder will rescore 20% of the videos to assess inter-rater reliability.

The same home visitor will interview families who are selected to continue to Phase 2 during a third home visit. Families from each group will be purposively sampled for maximum variation based on the change in the number of infant night wakings from baseline to outcome. Mothers and fathers will be interviewed as a family unit, rather than conducting individual interviews (78). Interviews will last up to 60 min and be digitally audio-recorded and transcribed verbatim. The semi-structured interview guides (Table S5 in Supplementary Material) include prompts that focus on capturing underlying relational and family processes in addition to eliciting individual family members’ perceptions and experiences (78, 79).

#### Quality Assurance, Monitoring, and Safety

Only the study investigators will monitor the study. No interim analysis is planned because the intervention is not expected to cause harm. Each completed study questionnaire will be reviewed for completeness upon participant submission. Participants who score in the clinical range on the EPDS (≥13 for mothers, ≥9 for fathers) will be flagged and encouraged to follow-up with their primary health care practitioner. Parents will be referred to the appropriate health care provider(s) or agencies if they experience severe distress at any point during the study.

The study protocol may be stopped or changed if the following safety concerns arise from either participants’ completed study questionnaires or the home visits: (1) concern for a participant’s potential to cause harm to self or others; (2) concerns of abuse or neglect of participants or their child; or (3) concern for the home visitor’s physical safety.

There are no known physical risks to participants taking part in this study. Participants may feel uneasy being video-recorded while playing with their baby, completing questionnaires, or answering questions during the interview. No audit is planned for this study.

## Anticipated Results

### Phase 1 Quantitative Analysis

Quantitative data will be analyzed using IBM SPSS Statistics 24.0 software. Data will be examined for frequency and patterns of missing data. If less than 20% of the data are missing, and if missing at random or completely at random, missing values will be imputed using multiple imputation (80). Descriptive statistics (means, SDs, frequencies, and percentages) will be used to describe the sample, and correlations between variables will be examined to identify potential covariates. Using the “intent to treat” principle, a RM-ANOVA will be used to detect differences between groups over time in mother and father reports of infant sleep patterns that are measured as continuous variables (night wakings, nocturnal wakefulness, and longest period of consolidated nighttime sleep). To control for Type I error that may result from multiple hypothesis testing, we will apply a Bonferroni correction during the analyses. Ordinal level infant sleep variables (sleep latency and parental perception of problematic sleep) will be analyzed using a Wilcoxon signed-rank test. If there are statistically significant differences between groups on the primary outcome, sub-analyses will identify which covariates have significant effects. Parent–child interactions scores for both mothers and fathers will be compared to normed scores available from the NCAST database.

### Phase 2 Qualitative (Thematic) Analysis

To align with Phase 1, the criterion variable for the thematic analysis will be the level of infant sleep disturbance, as indicated by the number of night wakings. To ensure rigor during the qualitative phase, Guba and Lincoln’s criteria of credibility, dependability, confirmability, transferability, and authenticity will be followed (81, 82). In addition, Braun and Clarke (83) provided a 15-point checklist that will be used as a benchmark throughout the process to enhance the credibility of the thematic analysis.

Braun and Clarke’s (83) six phases of thematic analysis, informed by Boyatzis’ (84) steps of inductive code development, will be used. These six phases include (1) familiarization with the data as a whole, (2) generating initial codes, (3) searching for themes, (4) reviewing themes, (5) defining and naming themes, and (6) producing the report. Although these processes are presented linearly, the actual analysis activities will progress together in an interdependent and fluid manner (83).

To develop the initial list of codes, a compare-and-contrast method in a subsample of transcripts will be used (84). Five transcripts will be chosen as a subsample and reviewed line-by-line (Elizabeth Keys) for key words or phrases within each subsample to develop codes based on similarities and differences across subsamples (84). A more experienced coder (Karen M. Benzies) will also code the subsample using the initial codes. Discrepancies in coding will provide discussion points regarding credibility, confirmability, and transferability. The remaining transcripts will then be coded and data will be organized into meaningful patterns (83, 84). NVivo 11 software will be used to assist in capturing the coded data extracts and managing the dataset.

Thematic maps will be used to articulate broad themes and thematic relationships, guided by the perspective that quantity does not necessarily dictate the creation of a theme, or its place in a hierarchy of themes (83). Paton’s (85) criteria of internal homogeneity and external heterogeneity will be used to discuss developing themes among a doctoral supervisory committee (Karen M. Benzies, Valerie Kirk, and Linda Duffett-Leger), as well as key clinical champions, to enhance dependability, transferability, and authenticity. As a form of member checking, each family who participated in the interviews will be sent a letter with thematic summaries. This letter will offer families an opportunity to review the codes, themes, and initial interpretations of the data that they provided during their interview. The letter will invite families to comment on the codes, themes, and interpretations.

The researchers will write a detailed analysis that articulates the name and essence of each theme to compile a final report (83), including a comprehensive analysis of how themes relate to one another. This qualitative component will provide rich insight into understanding parental experiences and perceptions that may influence the effectiveness of infant sleep interventions.

### Phase 3 Mixed Methods Integration

In Phase 3, the researchers will use the themes from the qualitative data to explain the quantitative results. During this phase, the researchers will explore how themes relate not only to the quantitative results but also how they relate to the theoretical research framework (41) from which the Play2Sleep intervention was developed. Understanding the explanatory mechanisms that contribute to the effectiveness of infant sleep interventions may help researchers and practitioners design and tailor future infant sleep interventions that are meaningful and effective for families. This approach will maximize the value of using a mixed methods approach to evaluate Play2Sleep.

### Clinical Implications

If effective, the effect sizes of Play2Sleep could be used to calculate accurate sample sizes in future studies that tailor and target Play2Sleep for different groups (i.e., premature infants, single-parent families). If Play2Sleep improves infant sleep, existing public health services may be able to integrate interventions that focus on parent–child interactions to improve infant sleep. Unlike behavioral-based extinction approaches (86), families of infants younger than 6 months could use Play2Sleep, and Play2Sleep may have additional application as an infant sleep disturbance prevention strategy. Including fathers in this research and using family interviews to explain the quantitative results makes this project unique and should result in evidence that better represents the complex and relational experiences of dual-parent families. Because this study includes the gold standard measure of parent–child interactions, it will generate the most precise information available to date regarding which specific domains of parent–child interactions are most important for infant sleep difficulties, and could be used to establish validity of the PCITS in a new population. The data collected on the comparison group will provide critical reference values for the effectiveness of the current public health nursing care for families coping with infant sleep difficulties. If Play2Sleep does not produce positive effects on night wakings, the comprehensive data may still be used to explore alternate hypotheses and research questions regarding the relationships between family health indicators and infant sleep disturbances.

### Limitations

Both convenience sampling and the subjective nature of parental report on infant sleep disturbances could be considered limitations. These limitations may lead to a recruitment bias as families experiencing milder difficulties may not perceive themselves to be eligible and those experiencing more severe sleep disturbances may not have the energy or motivation to participate. Inclusion of cohabiting mothers and fathers will limit the generalizability of results to single-parent and same-sex parent families. Furthermore, families enrolling in the study may be more motivated to achieve change in infant sleeping patterns, which may inflate improvements in infant sleep for both groups.

To minimize participatory burden, information on all possible covariates will not be collected. Specifically, no information on anxiety or perceived parental stress will be obtained. Similarly, although the expanded BISQ is a feasible and lower cost alternative to more objective sleep measures, such as actigraphy or polysomnography (87), this measure relies on subjective parental perceptions.

Another limitation of this study relates to the continuity and intimate involvement of the primary researcher in each phase, which may raise questions of bias. To increase transparency, the primary researcher will document activities that may promote or prevent bias. Other concerns for bias may relate to sampling and data collection techniques. For instance, although the naturalistic home environment typically enhances ecological validity for documenting parenting behaviors (88), it is possible that parents interact differently while being video-recorded interacting with their infant. Resource limitations necessitated the use of a single home visitor to conduct the initial home visits, when the experimental and comparison interventions are delivered. Using the same home visitor to deliver both interventions should contribute to intervention fidelity and help ensure that any differences between intervention and comparison groups are not due to differences in home visitors (i.e., ability to build rapport, clinical skill level). However, the use of a single home visitor precludes the ability to blind the home visitor to intervention allocation and it is possible that this may introduce bias in the study outcomes. For the qualitative interviews, it is important that families do not perceive the researcher to have a vested interest in a specific outcome, lest they avoid offering genuine perspectives that they perceive to conflict with the researcher’s needs. Moreover, data collection techniques may not capture all the extraneous or additional strategies families use to improve their infant’s sleep.

Lastly, in recent years, there has been a rapid increase in interest in promoting sleep that is observed in the vast increases in public interest in sleep, sleep research, proliferation of privatized pediatric sleep consultants, as well as the development and availability of consumer products. It is difficult to account for how these changes may influence the study results. We hope that asking parents, in both the baseline and outcome study questionnaires, to describe the resources, strategies, and services that they have already used in attempting to improve their infant’s sleep will help address this limitation and contextualize the results. If extraneous circumstances related to the increased commercialization of infant sleep were to significantly influence parental experiences of the intervention, we expect the family interviews to be useful in providing an in-depth understanding of these effects.

## Ethics and Dissemination

### Informed Consent and Institutional Review Boards

This protocol and study, including the informed consent forms, have received ethical approval from the University of Calgary Conjoint Health Research Ethics Board (REB15-2652), and will be renewed annually.

Individual informed consent will be obtained from each parent by ensuring that they understand the study purpose, what participation in the study entails, as well as the potential risks and benefits of participating in the study. Consent forms will be written at the eighth grade reading level. The consent form will be reviewed with each parent verbally over the phone prior to enrollment, along with an electronic copy provided to each parent *via* email. At the first home visit, the home visitor will obtain signed copies of the consent form from each parent after providing participants an additional opportunity to review information, ask additional questions, or obtain further clarification as needed.

At each home visit (baseline and outcome), families will receive a thank-you card containing a $10 gift card in recognition of the time and effort that families have allocated to the study. Each family who participates in the qualitative interviews will receive an additional $10 gift card.

### Confidentiality

Confidentiality of the data will be maintained by storing study data on a timed out, password-protected computer, in a secure room on campus. Back-up data files will be stored in locked filing cabinets in a separate room on password-protected hard drives. Only the research team will have access to the secure files. For the quantitative phase, data will be aggregated with no identifiers used when publishing results. To maintain confidentiality in the qualitative phase, pseudonyms will be assigned and used to replace any names used in the interview transcripts. Only the immediate study team members will have access to the study data.

### Dissemination

The research team anticipates publishing one primary paper that reports on the effectiveness of the Play2Sleep study on night wakings, using the qualitative findings to explain the quantitative results. All contributing authors will meet the International Committee of Medical Journal Editors suggested criteria for authorship (89). Findings from the study will be distributed and/or presented to local health care practitioners and agencies who supported study recruitment through a final report and a telehealth “breakfast and learn,” as well as to participants themselves. An online video that highlights the key findings will also be created for both parents and clinicians, and disseminated as part of a broader online social media campaign. Following study completion, de-identified data will be submitted to Secondary Analysis to Generate Evidence, a research and data facility operating under the authority of Policywise for Children & Families.^2^

## Notes

### Trial Status

On the date of manuscript submission, 54 mother–father–infant triads were enrolled in the trial. Based on current data, we anticipate needing to enroll 61 families to obtain complete data on 60 mother–father–infant triads.

## Ethics Statement

The study has been approved by an institutional review board (REB15-2652). Dissemination will include local and international presentations, peer-reviewed publications, and a social media campaign designed for parents and clinicians.

## Author Contributions

EK initiated the study and drafted the manuscript. KB, VK, and LD-L contributed to the study design and provided critical contributions to the manuscript. All authors approved the final manuscript.

## Conflict of Interest Statement

The authors declare that the research was conducted in the absence of any commercial or financial relationships that could be construed as a potential conflict of interest.

## Footnotes

^1^www.ncast.org (Accessed: January 10, 2018).

^2^https://policywise.com/initiatives/sage/ (Accessed: January 10, 2018).
