# Supplementary material for: Using Play to Improve Infant Sleep: A Mixed Methods Protocol to Evaluate the Effectiveness of the Play2Sleep Intervention
Source: Front Psychiatry. 2018 Apr 17;9:109. doi: 10.3389/fpsyt.2018.00109 (PMC5913340; doi:10.3389/fpsyt.2018.00109)
Supplement: Supplementary file 1 [file table_1.docx]

Supplementary Material

Using Play to Improve Infant Sleep: A Mixed Methods Protocol to Evaluate the Effectiveness of the Play2Sleep Intervention

Elizabeth Keys^1^, Karen Benzies^1*^, Valerie Kirk^2^, Linda Duffett-Leger^1^,

^1^Faculty of Nursing, University of Calgary, Calgary, Alberta, Canada

^2^Alberta Children's Hospital/University of Calgary, Calgary, Alberta, Canada.

*** Correspondence:** Dr. Karen Benzies: [benzies@ucalgary.ca](mailto:benzies@ucalgary.ca)

Table 5. *Semi-Structured Interview Guides*

| Intervention Group |
| --- |
| How has sleep been going for your family? (probe for elaboration of any sleep strategies/tools/resources used)What are some of your successes?Your concerns?Tell me about any changes in your parenting since participating in this study?Tell me about any changes in your family and relationships since participating in this study?Tell me about a time when you were most proud of the way you managed your baby’s sleep?Tell me about a time when you were most proud of the way your partner managed your baby’s sleep?Tell me about a time, that you used some of the pointers from when we watched the videos?How do you think you and your partner’s parenting and/or concerns about infant sleep may have changed since watching the videos?What was most helpful or interesting to you in this study?What was most difficult or uninteresting to you in this study?Tell me about watching the videos together.What was helpful?What was difficult?What would you change about the videos?What recommendations do you have if we decide to use this intervention with other new parents? (probe for timing of start, number of visits) |
| **Comparison Group** |
| - How has sleep been going for your family? (probe for elaboration of any sleep strategies/tools/resources used)   - What are some of your successes?   - Your concerns? - Tell me about any changes in your parenting since participating in this study? - Tell me about any changes in your family and relationships since participating in this study? - Tell me about a time when you were most proud of the way you managed your baby’s sleep? - Tell me about a time when you were most proud of the way your partner managed your baby’s sleep? - How do you think you and your partner’s parenting and/or concerns about infant sleep may have changed since the start of the study? - What was most helpful or interesting to you in this study? - What was most difficult or uninteresting to you in this study? - What recommendations do you have for sleep intervention with new parents? |
